# Effects of Phonological Consistency and Semantic Radical Combinability on N170 and P200 in the Reading of Chinese Phonograms

**DOI:** 10.3389/fpsyg.2021.603878

**Published:** 2021-07-09

**Authors:** Chun-Hsien Hsu, Ya-Ning Wu, Chia-Ying Lee

**Affiliations:** ^1^Institute of Cognitive Neuroscience, National Central University, Taoyuan City, Taiwan; ^2^Institute of Linguistics, Academia Sinica, Taipei, Taiwan; ^3^Research Center for Mind, Brain, and Learning, National Chengchi University, Taipei, Taiwan

**Keywords:** event-related potentials, N170, P200, visual word recognition, Chinese characters

## Abstract

Studies have suggested that visually presented words are obligatorily decomposed into constituents that could be mapped to language representations. The present study aims to elucidate how orthographic processing of one constituent affects the other and vice versa during a word recognition task. Chinese orthographic system has characters representing syllables and meanings instead of suffixation roles, and the majority of Chinese characters are phonograms that can be further decomposed into phonetic radical and semantic radical. We propose that semantic radical combinability indexed by semantic radicals and the effect of phonological consistency indexed by phonetic radicals would interact with each other during the reading of Chinese phonograms. Twenty-six right-handed native Chinese speakers were recruited to the study. Participants were presented with phonograms divided into four conditions following their semantic radical combinability (large vs. small) and phonological consistency (high vs. low). EEG signals were recorded throughout the covert naming task. Our results show that there is an interaction effect between phonological consistency and semantic radical combinability on the right hemisphere N170 activity while reading phonograms. Semantic radical combinability influenced the right hemisphere N170 during the process of low-consistency character reading but not high-consistency character reading. On the other hand, the left hemisphere N170 revealed a more significant activity during reading high-consistency characters and was not affected by radical combinability. In addition, while low-consistency characters revealed a larger P200 than high-consistency characters, the semantic radical combinability effect on P200 was only significant when participants were reading high-consistency characters but not low-consistency characters. These results provide new information about how ERPs are involved in word recognition within the context of interaction among orthographic and phonological dimensions.

## Introduction

An important aspect of visual word recognition is the ability to efficiently trigger the orthographic encoding mechanism in the left occipitotemporal cortex. Studies that measured N170/M170 components using electro- and magneto-encephalography (EEG and MEG), which allow for measuring brain activities in milliseconds, have also drawn attention to the left occipitotemporal cortex (Bentin et al., 1999; Tarkiainen et al., 1999; Maurer et al., 2008). N170/M170 components are electric/magnetic activities generated by the bilateral occipitotemporal cortex and peak at around 170 ms after the onset of words. Tarkiainen et al. (1999) has demonstrated that the amplitude of M170 activity in the left hemisphere is sensitive to the number of letter strings but not to the number of symbols. In functional magnetic resonance imaging (fMRI) studies, the left occipitotemporal cortex has been selective to orthographic stimuli (Dehaene and Cohen, 2011). EEG studies also have demonstrated that the left N170 response to word and letter strings is larger than that to symbols (Bentin et al., 1999; Maurer et al., 2008). In addition to the effect of orthographic versus non-orthographic stimuli on N170/M170, there is evidence that the morphological effects on these components take place (Solomyak and Marantz, 2010; Lewis et al., 2011; Fruchter et al., 2013). For example, Solomyak and Marantz (2010) have investigated the reading of suffixed words and found that the left M170 activity was positively associated with the transition probability between suffix and stems. Zweig and Pylkkänen (2009) also demonstrated that morphologically complex forms display larger amplitudes in the M170. This evidence indicated an obligatory process of decomposing complex words into morphological units in the early visual processing stage. Taken together, the evidence suggests that the reading-related N170 component reflects a unique orthographic encoding stage during visual word recognition.

On the other hand, the Chinese orthographic system does not have suffixation roles and has characters representing syllables and meanings. According to Myers (2019), 4,910 out of 5,908 traditional Chinese characters are unambiguously classified as phonograms which can be decomposed into a phonetic radical and a semantic radical (see Supplementary Figure). Among these phonograms, 63% have a semantic radical on the left and a phonetic radical on the right (SP characters), and 6% have an opposite layout (PS characters). The question then arose as to whether Chinese characters are decomposed into radicals during character recognition. Taft and Zhu (1997) addressed this question by defining radical frequency as the number of characters that share the same radical regardless of the radical position or function. Their behavioral experiments showed that only the frequency of the right radical had an impact on character recognition. The lexical decision time for characters with high frequency right radical was shorter than those with low frequency right radical. Furthermore, this was true only when the radical appeared on the right-hand side of the character. This finding implies that the right radical might be given priority over other positions in some way. However, according to the nature of phonograms described hereinabove, the interaction effect between radical position and radical function could have been easily confused with the radical function in the study of Taft and Zhu (1997). To clear up confusion, Feldman and Siok (1997) estimated the phonetic- and semantic-radical combinability by counting the number of phonograms sharing the same phonetic or semantic radicals and then manipulated radical position (left/right) and combinability (large/small) for both phonetic and semantic radicals in a character decision task. Feldman and Siok (1997) found facilitative combinability effects for both radicals; however, these effects were not reliable within their positions. For the phonetic radicals, the combinability effect was significant in both the left and right positions. For the semantic radicals, the combinability effect was significant only when the semantic radical was on the left. These findings suggest that the radical function should be considered when investigating the orthographic neighborhood size effect in Chinese characters.

In addition to behavioral studies, MEG and EEG studies have also demonstrated that Chinese phonograms are obligatorily decomposed into radicals during the reading of phonograms (Hsu et al., 2009, 2011). A MEG study on reading Chinese phonograms demonstrated orthographic neighborhood size on the M170 activity (Hsu et al., 2011). Phonograms with small semantic radical combinability elicit a larger M170 than those with large semantic radical combinability. In addition to radical combinability, previous studies have shown effects of phonological consistency on N170, in which phonological consistency is defined by the ratio of the number of characters with the same phonetic radical and the same pronunciation to the number of all characters with the same phonetic radical (Lee et al., 2007; Hsu et al., 2009). Taken together, these findings suggest that the representation of sublexical units is substantially involved in the early stages of visual word recognition.

Since Taft and Zhu (1997) and Feldman and Siok (1997), there has been a question as to whether phonetic and semantic radicals were processed independently or interdependently. To elucidate this question, we need to look at the evidence which implied that the semantic radical and the phonetic radical would interact with each other during the reading of Chinese phonograms. According to our analysis on Chinese phonograms drawn from the Academia Sinica Balanced Corpus (Huang and Chen, 1998), the combinability of phonetic radicals ranges from 1 to 20, and the combinability of semantic radicals ranges from 1 to 226. The enormous number of orthographic neighbors associated with semantic radicals might reduce its priority during character recognition; therefore, it is hypothesized that phonetic radicals prioritize semantic radicals. This assumption has been pointed out by Hsiao and Shillcock (2006) that the processing of radicals may skew to the phonetic radical in nature because it has high predictability on the characters. To evaluate this assumption, Hsiao et al. (2007) recorded ERPs while participants were performing a phonological recognition task. The target characters included SP and PS characters. The results showed that SP characters elicit larger N170 on the left-posterior sites than do those on the right-posterior sites, while PS characters elicit larger N170 on the right-posterior sites than do those on the left-posterior sites. Since the enhancement in the amplitude of N170 reflects the process of discriminating visual elements (Vogel and Luck, 2000), the opposite patterns of asymmetry at N170 between character types imply that Chinese readers attend to the site where the phonetic radical appears in the phonological recognition task.

Furthermore, it is also suggested that when the phonetic radical lacks character predictability, the processing of character recognition would skew to the semantic radical (Hsiao et al., 2006, 2007). This argument is supported by a repetitive transcranial magnetic stimulation (rTMS) study, in which participants performed semantic judgment tasks while semantic radical combinability was being manipulated; the stimuli were all SP characters (Hsiao et al., 2006). The study successfully replicated the facilitative radical combinability effect presented in the study of Feldman and Siok (1997) in the no rTMS condition. When rTMS was applied over the right occipital cortex, the facilitative radical combinability effect remained significant. However, applying rTMS over the left occipital cortex slowed down the processes of large semantic radical combinability characters. Presumably, rTMS over the left occipital cortex disturbed visual input projecting from the phonetic radical, which is on the right side of an SP character. This may help activate a set of candidates at the character level, consequently requiring a longer processing time on characters with large semantic radical combinability. This explanation implies that information of phonetic and semantic radicals might be processed interactively in some ways. Nevertheless, the evidence reviewed here was insufficient to address when phonetic radicals would interact with semantic radicals in Chinese character recognition. Previous ERP studies have demonstrated that N170 and P200 activities are associated with word form encoding processes and orthography-to-phonology transformation (Barber et al., 2004; Carreiras et al., 2005; Lee et al., 2007; Hsu et al., 2009). Therefore, we predict that the effect of semantic radical combinability and phonological consistency could interact with each other on early ERP components. In addition, Taft and Zhu (1997) have suggested that the right-hand radical is used to activate a set of candidates at the character level, and a selection is then made from these candidates based on the left-hand radical. If this is true for SP characters, there might be an interaction effect on the ERP component that reflects selection. Therefore, the present study evaluated the N400, reflecting the amount of lexical activity associated with orthographic neighbors during lexical assessment (Holcomb et al., 2002).

## Materials and Methods

### Participants

Twenty-six right-handed native Chinese speakers were recruited into this paid study. All participants were male college students with normal or corrected to normal vision. The Human Subject Research Ethics Committee/IRB of Academia Sinica, Taiwan, approved the current study. Due to insufficient valid trials (<12) after rejecting noisy EEG segments, one participant was excluded from the analyses after artifact rejection. The final analyses were performed on a total of 25 participants.

### Stimuli and Experimental Procedure

Eighty Chinese phonograms (listed in Supplementary Table 1) were selected as target characters from the Academia Sinica Balanced Corpus (Huang and Chen, 1998). All phonograms chosen comprised a semantic radical on the left and a phonetic radical on the right. Phonograms were then divided into four conditions: semantic radical combinability (large vs. small) and phonological consistency (high vs. low). Table 1 demonstrates the characteristics of each condition. Conditions were matched for the number of strokes [*F*_(3,76)_ = 0.37, *p* = 0.77], as well as character frequency [*F*_(3,76)_ = 0.32, *p* = 0.81] and phonetic radical combinability [*F*_(3,76)_ = 1.50, *p* = 0.22] Due to the lack of sufficient PS characters (6%), there would not be sufficient materials for an ERP experiment if the radical positions (i.e., using both PS and SP characters) were manipulated by consistency and semantic radical combinability simultaneously. Therefore, the present study mainly focused on reading SP characters, which are the majority of Chinese phonograms (63%). Every target character was paired with two probe characters that correspond with one another in their character frequency and the number of strokes. Between the two probes of each pairing, one would share the same pronunciation with their matching target character. Participants were instructed to sit at an approximate 60 cm distance from a monitor in an acoustically shielded room.

**Table 1:** Means and SDs (in parentheses) of parameters for stimuli in the experiment.

**Conditions**	**High consistency**		**Low consistency**	
	**Large combinability**	**Small combinability**	**Large combinability**	**Small combinability**
Examples	璞	幛	梢	糯
Consistency	0.97 (0.15)	0.99 (0.3)	0.27 (0.08)	0.28 (0.11)
Semantic radical combinability	136.15 (64.84)	18.35 (9.42)	106.45 (37.26)	14.35 (8.10)
Number of strokes	14.30 (4.11)	14.65 (3.31)	13.35 (4.12)	14.20 (4.54)
Character frequency	26.90 (11.70)	24.00 (26.56)	30.05 (10.42)	28.85 (27.99)
Phonetic radical combinability	6.50 (3.03)	5.30 (3.13)	7.45 (4.11)	6.85 (2.80)

**Figure 1:**
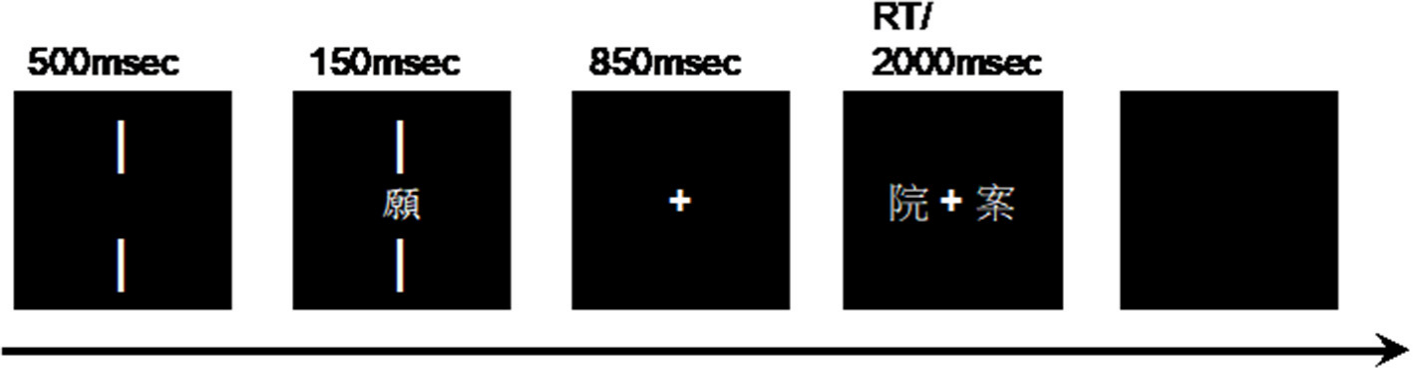
Schematic of the trial.

Figure 1 shows the summary of the trials. The trial began with the presentation of two vertical lines, one would be above, and the other would be below the center. Both lines would be presented simultaneously for 500 ms. The target character would then be displayed between the two vertical lines at the center of the screen for 150 ms. The size of the characters was approximately 1° × 1° visual angle. During the time, participants were required to maintain their gaze at the midpoint of the two lines while silently naming the target characters. The lines and target would then disappear from the monitor as a cross emerged at the center of it for 850 ms. Afterward, two probe characters would be presented at the left and the right of the cross simultaneously. If the left probe shared the same pronunciation as the target, participants should press a left button on a response pad with their left index finger. Similarly, if the right probe turned out to have the same pronunciation as the target character, participants should select the right button on a response pad with their right index finger. The positions of probe characters were counterbalanced among participants. After participants pressed a button, or after 2,000 ms without any response, a blank screen would show up for 1,600 ms to allow the participants to blink if necessary. The aim of this task was to ensure that participants knew the correct pronunciations of the target characters since processing the phonology of the targets without a covert naming response could cause muscular noise during the EEG recording. Moreover, previous studies manipulating semantic radical combinability were based on lexical decision tasks and semantic judgment tasks. We intend to examine how semantic radical combinability affects the reading of phonograms without emphasizing semantic knowledge. To ensure that participants were fixated on the designated fixation point, trials with target characters were mixed with 40 filler trials in the study of Hsiao et al. (2007). In filler trials, a 0.5° × 0.5° digit, instead of a character, was presented between the two lines where participants should have been fixating. Afterward, two digits would be presented at the left and the right of the cross simultaneously. Participants were required to select whether the left or the right digits was the same with the first digit during filler trials. For all participants, there were 30 practice trials and 120 randomized experimental trials (80 trials showing characters and 40 filler trials) in four test sessions. Participants were allowed to take breaks between sessions as long as they needed. The duration of the entire experiment was about 15 min. For trials showing target characters, the mean correctness of all the participants was 93% (ranging from 80 to 100%), and the mean response time was 668 ms (ranging from 475 to 957 ms). For filler trials, the mean correctness was 99% (ranging from 93 to 100%), and the mean response time was 436 ms (ranging from 369 to 542 ms).

### EEG Recording and ERPs Pre-processing

EEG was recorded from 64 Ag/AgCl electrodes (QuickCap, Neuromedical Supplies, Sterling, VI). A common vertex reference was placed between Cz and CPz. For further analysis, the data were re-referenced offline to the average left and the right mastoids. Vertical eye movements (VEOG) were recorded by a pair of electrodes located on the supra- and infra-orbital ridges of the left eye, while a pair of electrodes recorded horizontal eye movements (HEOG) placed lateral to the outer canthus of the left and right eyes. In addition, a ground electrode was placed anterior to Fz on the forehead. Electrode impedances were maintained below 5 KΩ. EEG signal was recorded and digitized continuously at a rate of 1,000 Hz. SYNAMPS2^®^ (Neuroscan, Inc., Walnut Creek, CA) amplifiers amplify recorded signals with a low-pass filter at DC−100 Hz for offline analysis. As for the offline analysis, the continuous wave was epoched with a pre-stimulus interval of 100 ms and a post-stimulus interval of 922 ms; the former was used for baseline correction. Moreover, trials were rejected if they were contaminated by eye movement or voltage variations larger than 60 mV. The data were then band-pass filtered at 10–30 Hz (zero phase shift mode, 12 dB). By averaging the corresponding trials, the ERPs of all conditions were then computed for each participant at every electrode site.

### Statistical Analyses

Four pairs of electrodes (P5/6, P7/8, PO5/6, and PO7/8) were selected from the left and right posterior areas, as the scalp from these regions is often associated with prominent reading-related N170 activities (Maurer et al., 2008; Hsu et al., 2009). For each electrode, condition, and subject, an N170 analysis was conducted by averaging the amplitude in a 50 ms window, centered at the negative peak within the time window from 100 to 200 ms. The repeated-measurement ANOVA (RM-ANOVA) analysis for N170 held four within-subject factors: consistency, semantic radical combinability, and hemispheres and electrodes. As for P200 analyses, nine electrodes (F1/3/5, FC1/3/5, and C1/3/5) were selected from the left anterior area since previous studies had reported consistency effect to be prominent in the region (Lee et al., 2007; Hsu et al., 2009). For each electrode, condition, and subject, P200 analyses were then conducted by averaging the amplitude in a 50 ms window centering at the positive peak within the time window from 100 to 300 ms. The mean amplitude was then averaged across electrodes so that the RM-ANOVA analysis for P200 consisted of two within-subject factors: consistency and semantic radical combinability. For N400 analyses, the consistency and combinability effects were compared using the mean amplitude from the time windows of interest (300–450 ms). Since the components were distributed over the entire scalp, the analyses of N400 were then separated into a midline analysis and a lateral analysis. Five electrodes (FZ, FCZ, CZ, CPZ, and PZ) were selected to be the electrode variable for the midline N400. Another eight electrodes (F3/4, FC3/4, C3/4, CP3/4) were also selected as the variable for the lateral N400. The midline N400 was tested by a three-way RM-ANOVA that included character consistency, phonetic combinability, and electrodes as within-subject factors. On the other hand, the lateral N400 was tested by a four-way RM-ANOVA, which included character consistency, phonetic combinability, hemisphere, and electrodes as within-subject factors.

To correct the violations of sphericity associated with repeated measures, the Greenhouse-Geisser adjustment to the degrees of freedom was applied for each RM-ANOVA. The corrected *p*-values for all the F tests with more than one degree of freedom in the numerator were reported. The *post-hoc* tests were performed using Tukey's procedure.

## Results

### N170

**Figure 2:**
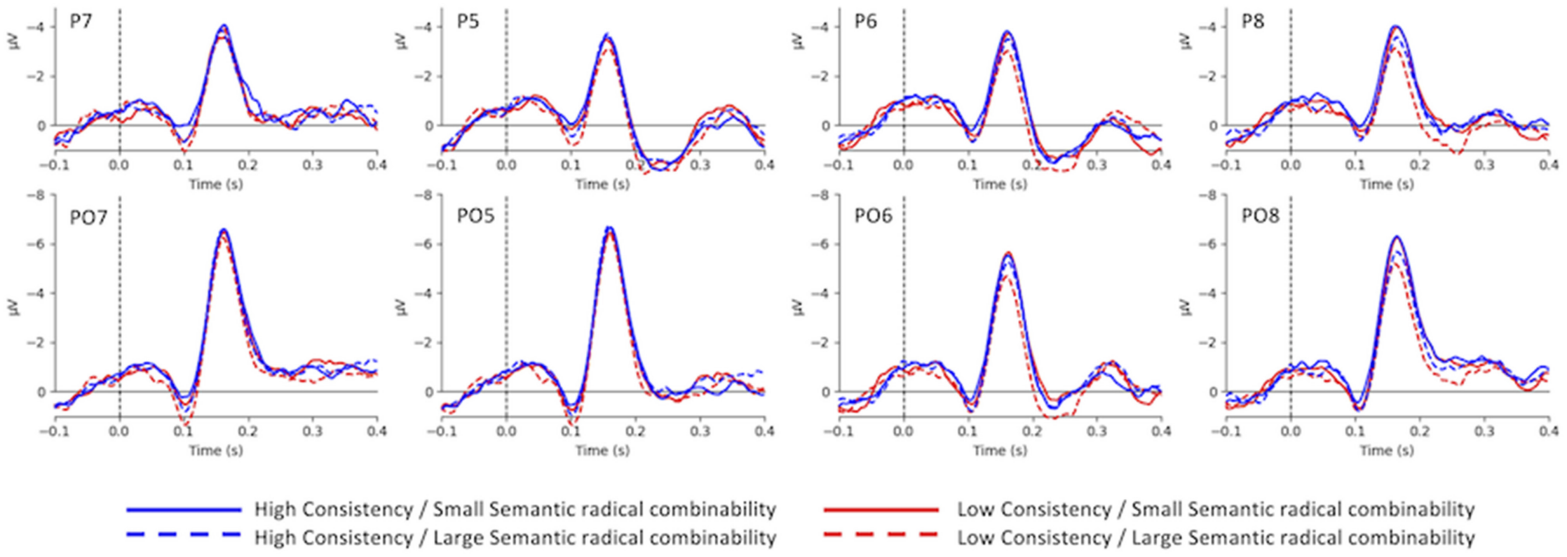
The grand-average waves of N170.

**Figure 3:**
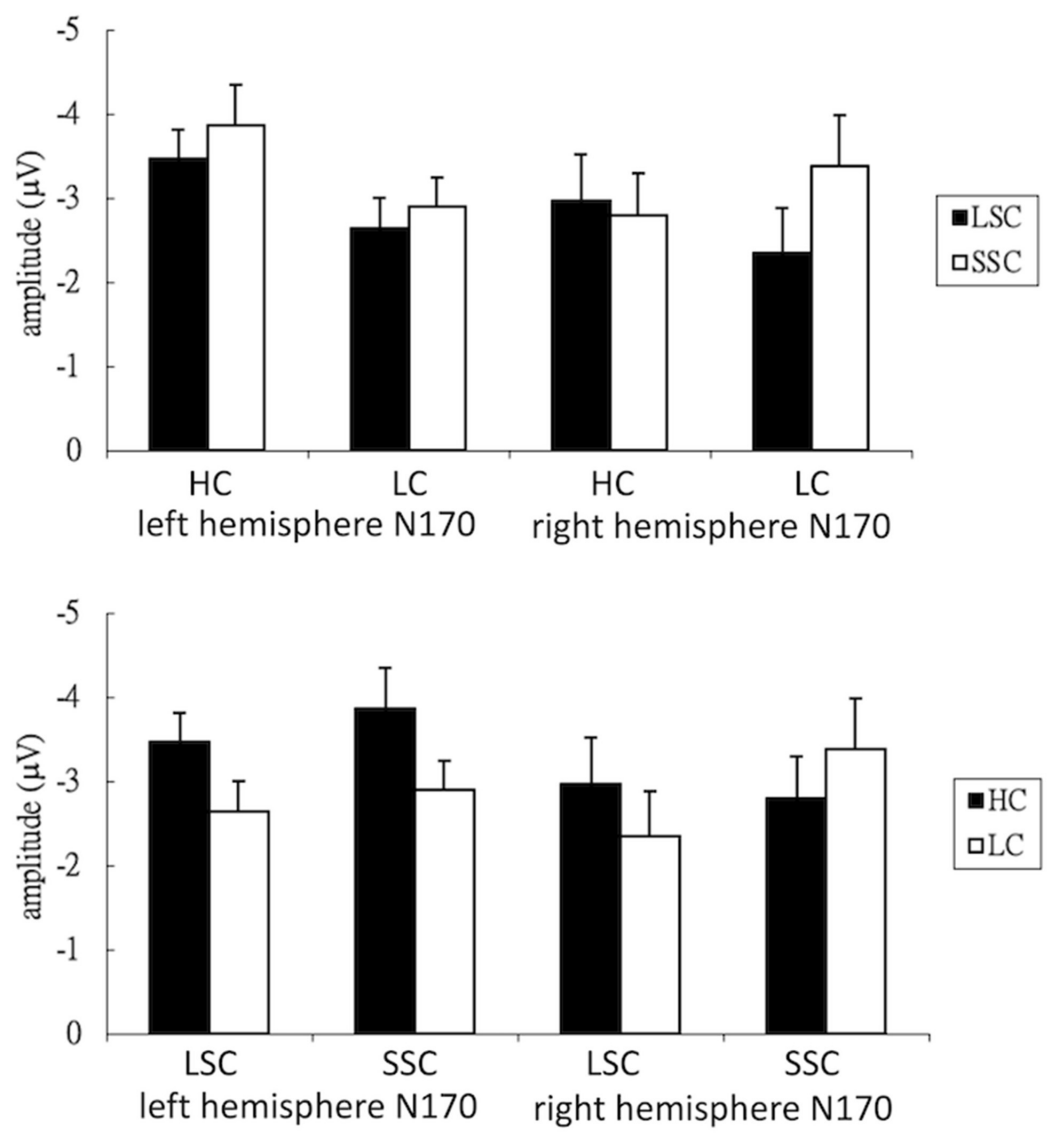
Bar plots of the mean N170 response. HC, high consistency; LC, low consistency; LSC, large semantic radical combinability; SSC, small semantic radical combinability; left, left hemisphere; right, right hemisphere.

Figure 2 demonstrates the grand-average waves for each condition in each electrode of interest selected for N170 analysis. Main effects were significant for both consistency [*F*_(1,24)_ = 9.77, *p* < 0.01, $\eta_{p}^{2}$ = 0.29] and semantic radical combinability [*F*_(1,24)_ = 2.90, *p* < 0.05, $\eta_{p}^{2}$ = 0.11]. In other words, high-consistency characters triggered larger N170 than low-consistency characters; and small semantic radical combinability characters triggered larger N170 than large semantic radical combinability characters. Additionally, there was a significant three-way interaction among consistency, semantic radical combinability and hemisphere [*F*_(1,24)_ = 13.16, *p* < 0.01, $\eta_{p}^{2}$ = 0.35]. *Post-hoc* tests revealed that (Figure 3) small semantic radical combinability characters triggered larger right hemisphere (RH) N170 than large semantic radical combinability characters; this effect was significant in the reading of low-consistency characters [*F*_(1,24)_ = 17.78, *p* < 0.001, $\eta_{p}^{2}$ = 0.43] but not in the reading of high-consistency characters [*F*_(1,24)_ = 0.70, *p* > 0.1, $\eta_{p}^{2}$ = 0.03]. On the other hand, simple main effects of semantic radical combinability were not significant on the LH N170 (ps > 0.05).

Regarding the simple main effects of phonological consistency, left hemisphere (LH) N170 to high-consistency characters was larger than that to low-consistency characters, and this effect was significant in the reading of small semantic radical combinability characters [*F*_(1,24)_ = 18.19, *p* < 0.001, $\eta_{p}^{2}$ = 0.43] and was marginally significant in the reading of large semantic radical combinability characters [*F*_(1,24)_ = 4.25, *p* = 0.05, $\eta_{p}^{2}$ = 0.15]. Lastly, for RH N170, small and large semantic radical combinability characters showed an opposite patterns of consistency effects. For large semantic radical combinability characters, high-consistency characters triggered larger RH N170 than low-consistency characters [*F*_(1,24)_ = 6.67, *p* < 0.05, $\eta_{p}^{2}$ = 0.22]. For small semantic radical combinability characters, low-consistency characters triggered larger RH N170 than high-consistency characters [*F*_(1,24)_ = 6.10, *p* < 0.05, $\eta_{p}^{2}$ = 0.20]. The four-way interaction between consistency, semantic radical combinability, hemisphere and electrodes was significant [*F*_(3,72)_ = 30.71, *p* < 0.001, $\eta_{p}^{2}$ = 0.56]. Simple main effects of the four-way interaction were listed in Supplementary Table 2.

### P200

**Figure 4:**
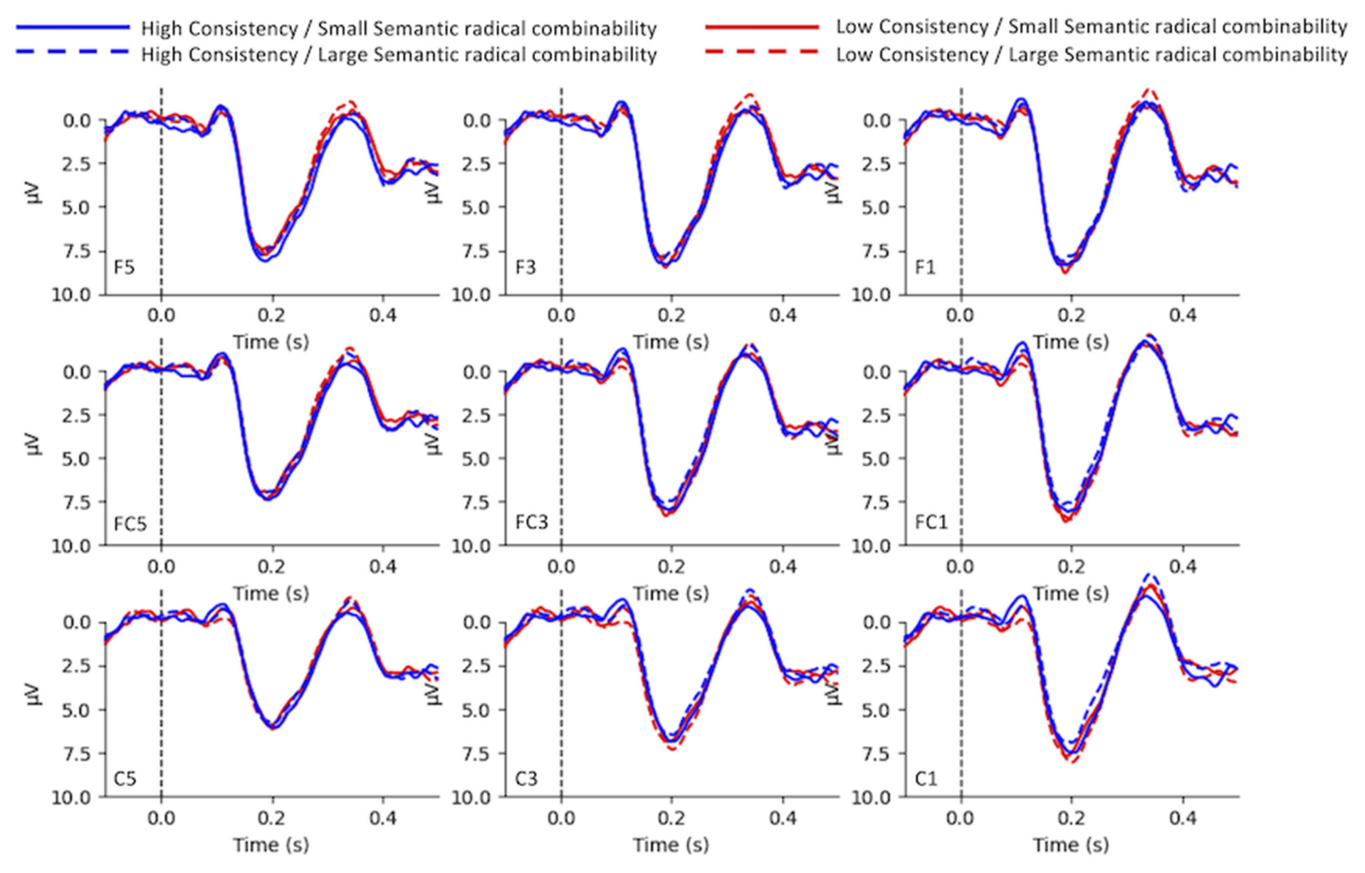
The grand-average waves of P200.

**Figure 5:**
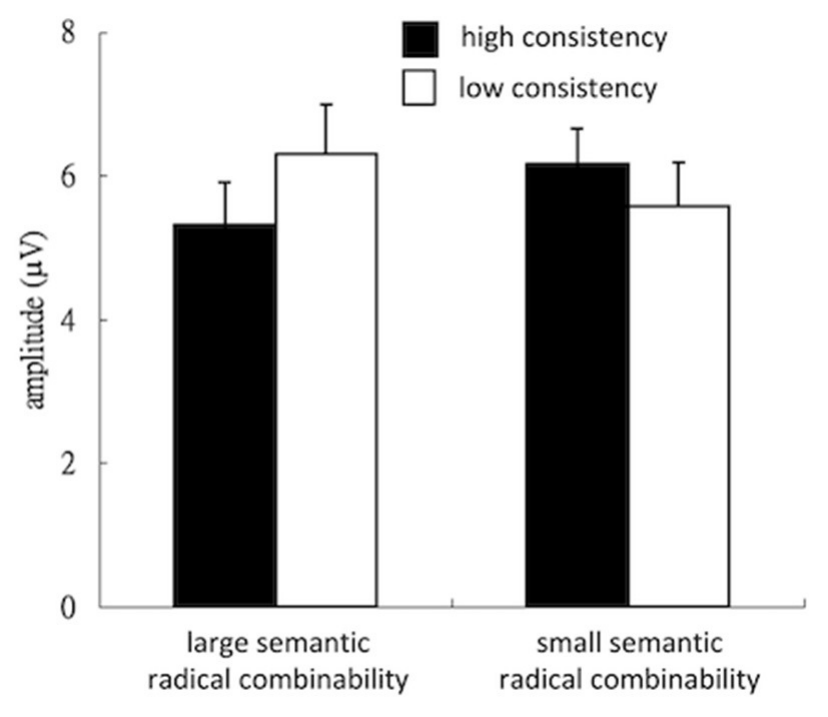
Bar plots of the mean P200 response. HC, high consistency; LC, low consistency; LSC, large semantic radical combinability; SSC, small semantic radical combinability.

Figure 4 illustrates the grand-average waves in electrodes of interests. Neither consistency [*F*_(1,24)_ = 0.55, *p* > 0.1, $\eta_{p}^{2}$ = 0.02] nor semantic radical combinability [*F*_(1,24)_ = 0.05, *p* > 0.1, $\eta_{p}^{2}$ = 0.002] showed significant main effects. The interaction among consistency and semantic radical combinability was significant [*F*_(1,24)_ = 7.85, *p* < 0.01, $\eta_{p}^{2}$ = 0.25]. *Post-hoc* tests revealed that (Figure 5) the simple main effect of consistency was significant in characters with large semantic radical combinability [*F*_(1,24)_ = 6.19, *p* < 0.05, $\eta_{p}^{2}$ = 0.21], but not in those with small semantic radical combinability [*F*_(1,24)_ = 2.17, *p* > 0.1, $\eta_{p}^{2}$ = 0.08]. In other words, characters with low consistency/large semantic radical combinability led to larger positive P200 than those with high consistency/large semantic radical combinability. Moreover, the simple main effect of semantic radical combinability was significant in characters with high consistency [*F*_(1,24)_ = 4.57, *p* < 0.05, $\eta_{p}^{2}$ = 0.16], but not in those with low consistency [*F*_(1,24)_ = 3.33, *p* > 0.05, $\eta_{p}^{2}$ = 0.12]. That is to say, characters with high consistency/small semantic radical combinability triggered larger positive P200 than those with high consistency/large semantic radical combinability.

### N400

**Figure 6:**
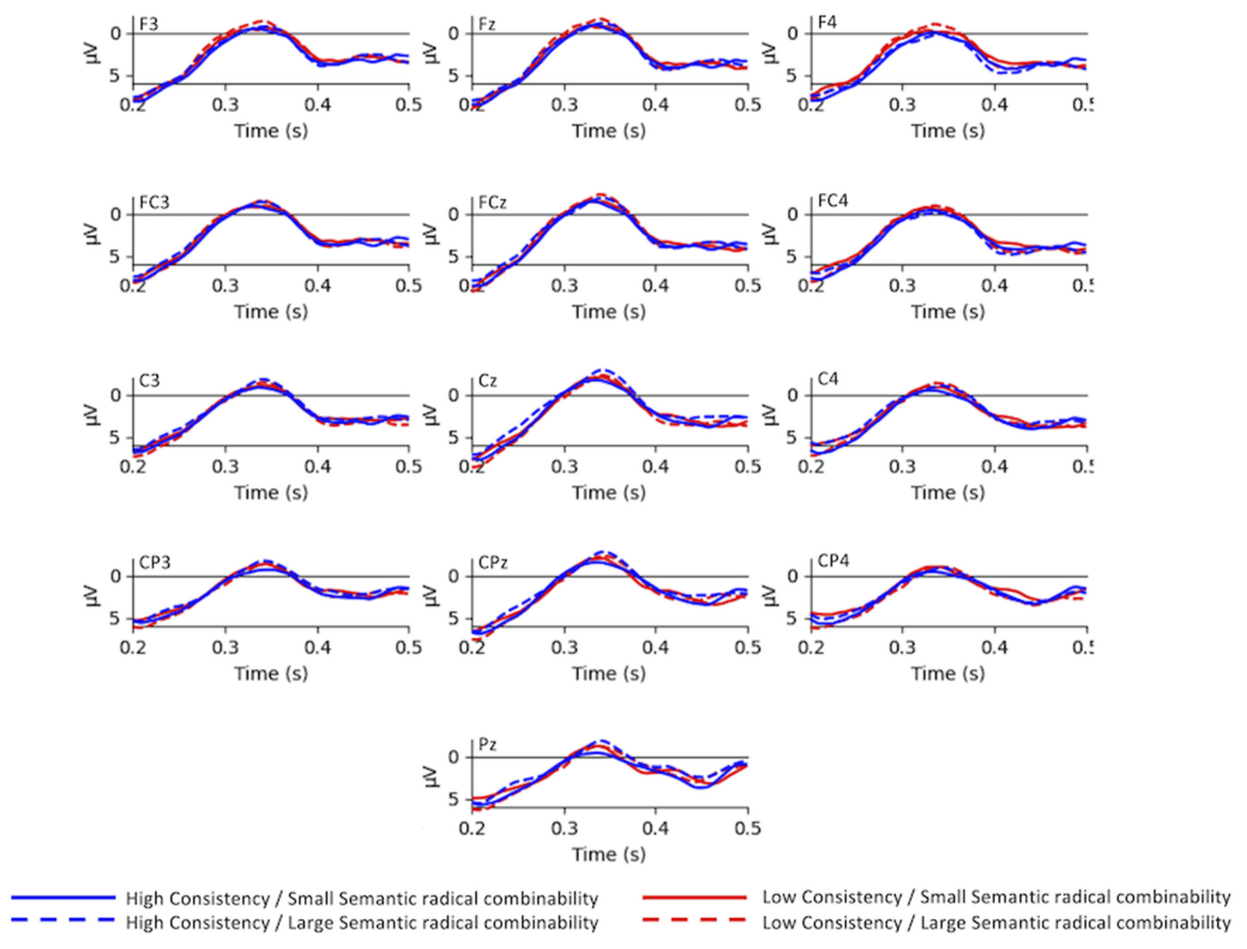
The grand-average waves of N400.

Figure 6 demonstrates the grand-average waves for N400 analysis. Neither the midline analysis nor the lateral analysis showed significant main effects of consistency [midline: *F*_(1,24)_ = 0.10, *p* > 0.1, $\eta_{p}^{2}$ = 0.0004; lateral: *F*_(1,24)_ = 0.57, *p* > 0.1, $\eta_{p}^{2}$ = 0.02] or semantic radical combinability [midline: *F*_(1,24)_ = 0.39, *p* > 0.1, $\eta_{p}^{2}$ = 0.02; lateral: *F*_(1,24)_ = 0.001, *p* > 0.1, $\eta_{p}^{2}$ = 0.00]. The interaction among consistency and semantic radical combinability was not significant [midline: *F*_(1,25)_ = 0.310, *p* > 0.1, $\eta_{p}^{2}$ = 0.01; lateral: *F*_(1,25)_ = 0.06, *p* > 0.1, $\eta_{p}^{2}$ = 0.002]. The lateral analysis revealed a significant interaction among consistency and electrodes [*F*_(3,72)_ = 4.25, *p* < 0.05, $\eta_{p}^{2}$ = 0.15]. Post- hoc tests showed that low-consistency characters triggered larger N400 than high consistency characters in anterior electrodes [electrode F3/4: *F*_(1,24)_ = 26.09, *p* < 0.001, $\eta_{p}^{2}$ = 0.27; electrode FC3/4: *F*_(1,24)_ = 6.60, *p* < 0.05, $\eta_{p}^{2}$ = 0.08] and such consistency effect was not significant in posterior electrodes [electrode C3/4: *F*_(1,24)_ = 0.42, *p* > 0. 1, $\eta_{p}^{2}$ = 0.006; electrode CP3/4: *F*_(1,24)_ = 0.75, *p* > 0.1, $\eta_{p}^{2}$ = 0.01].

## Discussion

The current study aimed to examine whether early ERP activity is sensitive to the function of constituents of word forms. The question was answered by evaluating the ERP signature of the interaction of phonological consistency and semantic radical combinability for Chinese phonograms. Based on the behavioral findings (Hsiao et al., 2006) and ERP results (Hsiao et al., 2007), we predict that the activity of ERPs associated with orthographic and phonological processing would demonstrate the interaction between the effect of semantic radical combinability indexed by semantic radicals and the effect of phonological consistency indexed by phonetic radicals during the reading of Chinese phonograms.

The present results replicated the effect of phonological consistency on N170 (Lee et al., 2007; Hsu et al., 2009), exhibiting an enhanced response to characters with high consistency. Furthermore, semantic radical combinability did not influence the LH N170. The activity of LH N170 appears to track the degree of the saliency of phonetic radicals in phonograms since the amplitude enhancement of N170 is known to reflect the process of visual element discrimination (Vogel and Luck, 2000). The results above suggest that phonetic radical might prioritize the semantic radical during the orthographic encoding process supported by the left hemisphere. While the results did not show any semantic radical combinability on LH N170, there was an interaction effect of phonological consistency and semantic radical combinability on RH N170 as predicted. Implications on the applicable rules of N170 in visual word recognition are discussed below.

In general, the results of this study support the idea that RH N170 and LH N170 concurrently play different roles during the encoding of word form. Perfetti and colleagues have shown that phonological representation is activated rapidly and early in Chinese word recognition (Perfetti and Zhang, 1995; Perfetti and Tan, 1998; Tan and Perfetti, 1999). In the present results, for LH N170, the phonological consistency effect at the N170 time window also implies early activations of phonological representations at the sublexical processing level. The timing of the phonological consistency effect was compatible with previous studies showing effects of phonological regularity of Chinese phonograms on N170 in lexical decision tasks, delayed naming tasks, and one-back repetition detection tasks (Yum et al., 2014; Yum and Law, 2021). Overall, the results mentioned above indicate that the mapping of orthography to phonology influences the earlier stages of orthographic processing (Goswami and Ziegler, 2006). On the other hand, the semantic radical combinability effect on RH N170 replicated the findings of Hsu et al. (2011), which demonstrated that RH M170 is associated with the neural representations of semantic radicals. Furthermore, the simple main effect of semantic radical combinability on RH N170 activity was significant in reading low-consistency characters but not in the reading of high-consistency characters. An interaction effect implies that the activation of semantic radicals varies as a function of phonological consistency in the N170 time window. According to Hsiao (2011), characters with low phonological consistency have a less skewed information distribution than those with high phonological consistency. Thus, participants may pay more attention to the semantic radical on the left for characters with a lower phonological consistency, leading to an increased activation/response amplitude in the RH N170. A similar explanation seems to account for the finding that the phonological consistency effect on RH N170 only manifests in the reading of large semantic radical combinability but not in the reading of small radical combinability. Taken together, we speculated that during the encoding of words indexed by N170, the phonetic radical might have been given priority over the semantic radical. The sublexical representation of semantic radical seems to be activated depending on the phonological consistency and semantic radical combinability.

One might ask why the RH N170 but not the LH N170 showed an interaction between radicals. A possible explanation for such results could be tied to the phonological mapping hypothesis of the N170 word effect (Maurer and McCandliss, 2007), which suggested that the left lateralization of the reading-related N170 may reflect the involvement of sublexical phonological representation during visual word recognition. For characters with low consistency and small semantic radical combinability, their sublexical representation could be activated in a way that prioritizes the use of semantic radicals, therefore resulting in the interaction between phonetic and semantic radicals in RH N170.

In addition to N170, the present study demonstrated an interaction effect between consistency and semantic radical combinability on P200. Low-consistency characters revealed a larger P200 than high-consistency characters. Phonological consistency on P200 suggests that this component could reflect the access of phonological representation, which is associated with sublexical units such as graphemes and phonetic radicals (Barber et al., 2004; Carreiras et al., 2005). Hsu et al. (2014) used source analysis techniques in combination with MEG and have found consistent effects in the activity of the left inferior parietal cortex at the P200 time window. Previous brain imaging studies have indicated that the left inferior parietal cortex supports the integration of orthography and phonology in visual word recognition (Pugh et al., 2000; Booth et al., 2003; Clark and Wagner, 2003; Tan et al., 2005). Therefore, the consistency effect in P200 suggests that the phonetic radical activates the phonological units, while low-consistency characters generate more activity at the phonological level. Moreover, the present results also showed that such consistency effect on P200 was significant in reading large semantic radical combinability characters but not in the reading of small semantic radical combinability characters. This finding suggests that the predicted interaction between radical units would commence not only in the encoding of word form but also in the activation of phonological representation.

Regarding the semantic radical combinability effect on P200, ERP studies of orthographic neighborhood size effects and syllable frequency effects in the reading of alphabetic words have demonstrated a consistent finding that the number of orthographic neighbors associated with a given word is inversely correlated with the amplitude of P200 (Barber et al., 2004; Carreiras et al., 2005). This explanation seems to account for the present finding that characters with few orthographic neighbors would reveal large P200. Additionally, Hsiao et al. (2006) have speculated that semantic radical combinability would vanish when the processes of the phonetic radical are interrupted. However, the present results demonstrated a similar finding that simple main effects of semantic radical combinability on P200 were modulated by phonological consistency. The semantic radical combinability effect on P200 was only significant in reading high-consistency characters but not in the reading of low-consistency characters.

Finally, the present study demonstrated that the N400 to low-consistency characters were larger than that to high-consistency characters and that this consistency effect on N400 was located in the anterior scalp. In brain imaging studies, it has been proposed that N400 is generated by a distributed network in the brain (Lau et al., 2008), and among these regions, the inferior prefrontal cortex has been suggested to be related to semantic elaboration and the selection of semantic knowledge (Thompson-Schill et al., 1997, 1999). Hence, reconciliation is needed for the hypothesis of semantic processing with the current finding that the reading of low-consistency characters revealed a large N400 in the frontal scalp. Furthermore, simulation and behavioral studies have suggested that the reading of low-consistency words would benefit from the activation of semantic representations when phonological information computation is in a rather slow and error-prone state (Strain et al., 1995, 2002; Woollams et al., 2007). Accordingly, the present consistency effect on N400 implies the possibility that participants were accessing the semantic information while reading low-consistency phonograms.

This study has some potential limitations. First, it should be noted that we based our experiment design on the findings of Hsiao et al. (2006, 2007). Referring to their methods, we selected only male participants accordingly as men were shown to exhibit more significant patterns of left lateralization. In the future, we would be interested in looking into the impact of factors such as gender and language fluency on ERP components. Second, this study only employed SP characters (semantic radical on the left and phonetic radical on the right). Further research could test whether PS characters also demonstrate the same effect as SP characters on N170. Considering that the number of PS characters is too small (6%) for factorial design experiments, the single-trial analysis could be used for future studies. In conclusion, the evidence from this study suggests that phonetic radical and semantic radicals are processed interactively in the stages of orthographic encoding and phonological activation—indexed by N170 and P200, respectively.

## Data Availability Statement

The datasets generated for this study are available on request to the corresponding author.

## Ethics Statement

The studies involving human participants were reviewed and approved by Human Subject Research Ethics Committee/Institutional Review Board of Academia Sinica, Taiwan. All participants provided their written informed consent to participate in this study.

## Author Contributions

C-HH contributed to study conception, experimental design, collecting data, performing the statistical analysis, and writing the first draft of the manuscript. Y-NW revised and proofread the manuscript. C-YL contributed to the study conception and discussion of the results. All authors contributed to the article and approved the submitted version.

## Conflict of Interest

The authors declare that the research was conducted in the absence of any commercial or financial relationships that could be construed as a potential conflict of interest.
