# Supplementary material for: Effects of Phonological Consistency and Semantic Radical Combinability on N170 and P200 in the Reading of Chinese Phonograms
Source: Front Psychol. 2021 Jul 9;12:603878. doi: 10.3389/fpsyg.2021.603878 (PMC8299066; doi:10.3389/fpsyg.2021.603878)
Supplement: Supplementary file 1 [file Table_1.docx]

**Supplementary Figure.** An example of a phonogram and its semantic/phonetic radicals

**
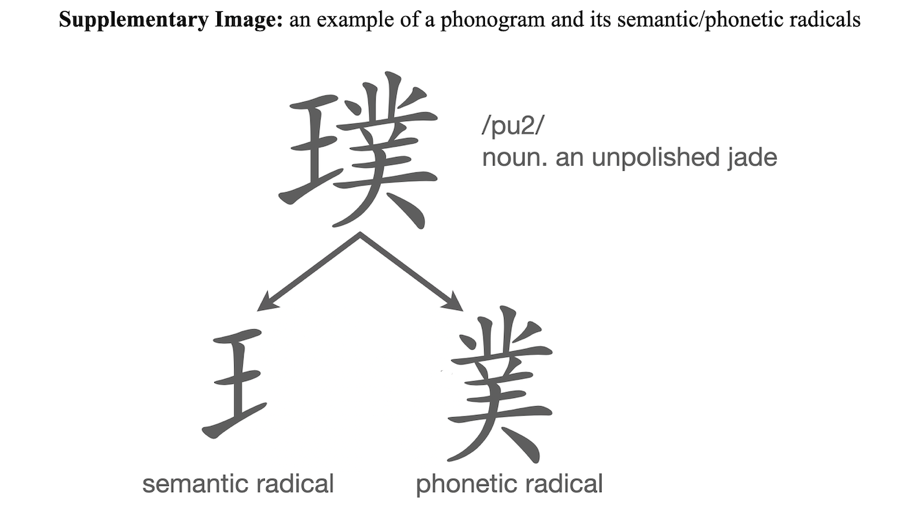
**

**Supplemental Table 1.** Lists of target characters

| High Consistency  Large Semantic Radical combinability | 璞 鐐 瀘 瀝 蠣 檸 饑 嘻 嗷 嗚  堰 偎 渙 渝 蜿 絹 烽 洌 蛀 伕 |
| --- | --- |
| High Consistency  Small Semantic Radical combinability | 幛 殭 殤 朦 穠 鰱 糠 幔 轅 稼  徨 鯧 艋 弒 鞍 祐 舫 軛 耘 韌 |
| Low Consistency  Large Semantic Radical combinability | 梢 臍 璿 鐲 譎 慚 縷 揀 悵 鋅  懺 悖 絢 鉀 柚 怵 玷 絆 眨 眈 |
| Low Consistency  Small Semantic Radical combinability | 糯 殮 韃 幢 轆 犒 鰭 徬 鞦 耦  鯨 竦 皓 聒 弧 舵 歿 靴 弛 畝 |

**Supplemental Table 2.** Simple main effects of consistency and semantic radical combinability on N170

| effects of phonological consistency (high minus low) | | | | |
| --- | --- | --- | --- | --- |
| channels | large semantic radical combinability | | small semantic radical combinability | |
|  | t statistics | p values | t statistics | p values |
| (left hemisphere) |  |  |  |  |
| P5 | **-2.757** | **.005** | 1.413 | .085 |
| PO5 | -0.516 | .305 | **-12.151** | **< .0001** |
| P7 | -.164 | .435 | -0.807 | .213 |
| PO7 | **-2.310** | **.015** | -0.340 | .368 |
| (right hemisphere) |  |  |  |  |
| P6 | -0.827 | .208 | -0.537 | .298 |
| PO6 | **-4.276** | **.0001** | **2.198** | **.019** |
| P8 | -1.193 | .122 | 0.101 | .460 |
| PO8 | -0.904 | .180 | **5.123** | **< .0001** |
|  | | | | |
| effects of semantic radical combinability (large minus small) | | | | |
| channels | high consistency | | Low consistency | |
|  | t statistics | p values | t statistics | p values |
| (left hemisphere) |  |  |  |  |
| P5 | **3.326** | **.001** | **7.607** | **<.0001** |
| PO5 | 0.558 | .291 | **-11.387** | **<.0001** |
| P7 | 0.551 | .293 | -0.108 | .457 |
| PO7 | -0.102 | .459 | 1.921 | .187 |
| (right hemisphere) |  |  |  |  |
| P6 | 1.085 | .144 | 1.382 | .089 |
| PO6 | -0.234 | .408 | **6.413** | **<.0001** |
| P8 | -1.324 | .098 | 0.004 | .498 |
| PO8 | **-1.924** | **.033** | **4.263** | **.0001** |
